# Platinum(II) Complexes with Bulky Disubstitute Triazolopyrimidines as Promising Materials for Anticancer Agents

**DOI:** 10.3390/ma13235312

**Published:** 2020-11-24

**Authors:** Iwona Łakomska, Dariusz Śmiłowicz, Mateusz Jakubowski, Jerzy Sitkowski, Andrzej Wojtczak

**Affiliations:** 1 Faculty of Chemistry, Nicolaus Copernicus University in Toruń, Gagarina 7, 87-100 Toruń, Poland; mjakubowski@umk.pl (M.J.); awojt@chem.umk.pl (A.W.); 2 Inorganic Chemistry I—Bioinorganic Chemistry, Ruhr-University Bochum, Universitätsstraße 150, 44801 Bochum, Germany; dariusz.smilowicz@rub.de; 3 Institute of Organic Chemistry, Polish Academic of Science, Kasprzaka 44/52, 01-224 Warszawa, Poland; j.sitkowski@nil.gov.pl; 4 National Medicines Institute, Chełmska 30/34, 00-725 Warszawa, Poland

**Keywords:** platinum(II) complexes, dicarboxylato ligands, triazolopyrimidines, multinuclear NMR, lipophilicity, in vitro cytotoxicity

## Abstract

Herein, we present dicarboxylate platinum(II) complexes of the general formula [Pt(mal)(DMSO)(L)] and [Pt(CBDC)(DMSO)(L)], where L is dbtp 5,7-di*tertb*utyl-1,2,4-triazolo[1,5-*a*]pyrimidine) or ibmtp (7-isobutyl-5-methyl-1,2,4- triazolo[1,5-*a*]pyrimidine), as prospective prodrugs. The platinum(II) complexes were synthesized in a one-pot reaction between *cis*-[PtCl_2_(DMSO)_2_], silver malonate or silver cyclobutane-1,1-dicarboxylate and triazolopyrimidines. All platinum(II) compounds were characterized by FT-IR, and ^1^H, ^13^C, ^15^N and ^195^Pt NMR; and their square planar geometries with one monodentate N(3)-bonded 5,7-disubstituted-1,2,4-triazolo[1,5-*a*]pyrimidine, one S-bonded molecule of dimethyl sulfoxide and one O,O-chelating malonato (**1**, **2**) or O,O-chelating cyclobutane-1,1-dicarboxylato (**3**, **4**) was determined. Additionally, [Pt(CBDC)(dbtp)(DMSO)] (**3**) exhibited (i) substantial in vitro cytotoxicity against the lung adenocarcinoma epithelial cell line (A549) (IC_50_ = 5.00 µM) and the cisplatin-resistant human ductal breast epithelial tumor cell line (T47D) (IC_50_ = 6.60 µM); and (ii) definitely exhibited low toxicity against normal murine embryonic fibroblast cells (BALB/3T3).

## 1. Introduction

Inert square planar Pt(II) drugs currently used in the chemotherapy of some cancers suffer from lack of selectivity, toxicity and poor aqueous solubility, and in many cases acquired or inherent resistance also develops during treatments [1,2,3,4,5,6,7]. To overcome these drawbacks, there is an urgent need to find more selective and low-concentration but effective metal-based chemotherapeutics with improved pharmacological properties [8,9,10] and far fewer side effects than those of current drugs. These new strategies are currently in progress and often take advantage of the differences between healthy and cancer cells [11].

The main approach in the rational design of platinum(II) complexes focuses on modifications in either the carrier or the leaving ligands [12,13,14]. It is well known that the presence of the dicarboxylato moiety in the coordination sphere enhances the aqueous solubility of platinum(II) complexes [15], reduces side effects and increases the efficiency of platinum(II) anticancer drugs [16,17,18], and does not affect the mechanism of activation and interaction with DNA bases [19]. Dicarboxylato often undergoes chelation, producing five- or six-membered rings with platinum(II) ions, which improves complex stability compared to those of chloride analogues and decreases the interactions with sulfur-containing molecules, such as glutathione, methionine and cysteine, in the bloodstream.

Currently, attention has been focused on the synthesis of complexes with aromatic N-donor molecules as carrier ligands. Dicarboxylato platinum(II) compounds with heterocyclic derivatives are of interest due to their antiproliferative activity and may represent a new class of promising anticancer prodrugs. Recently, oxalate platinum(II) complexes involving 7-azaindole halogeno-derivatives (nHaza), namely, [Pt(ox)(3ClHaza)_2_] and [Pt(ox)(3BrHaza)_2_], where 3ClHaza symbolizes 3-chloro-7-azaindole and 3BrHaza symbolizes 3-bromo-7-azaindole, have been synthesized [20]. Other oxalate platinum(II) complexes contain derivatives of the plant hormone kinetin (N6-furfuryladenine), namely, [Pt(ox)(L_1_)] and [Pt(ox)(L_2_)], where L_1_-2-chloro-N6-furfuryl-9-isopropyladenine and L_2_-2-chloro-N6-(5-methylfurfuryl)- 9-isopropyladenine [21]. Additionally, our previous initial in vitro antiproliferative activity tests proved that symmetric malonato platinum(II) complexes of the general formula [Pt(mal)(L)_2_], where L is 7-isobutyl-5-methyl-1,2,4-triazolo[1,5-*a*]pyrimidine (ibmtp) and 5,7-di*tert*butyl- 1,2,4-triazolo-[1,5-*a*]pyrimidine (dbtp), exhibit excellent in vitro activity against the cisplatin-resistant human ductal breast epithelial tumor cell line (T47D) and against a lung adenocarcinoma epithelial cell line (A549). In vitro antiproliferative studies showed that symmetrical [Pt(mal)(ibmtp)_2_] (3.44 µM) and [Pt(mal)(dbtp)_2_] (2.66 µM) complexes exhibit higher in vitro activity against T47D and A549 cell lines than cisplatin [22]. These results are greatly promising, but their toxic activity against normal cells blocked their medical application. Therefore, researchers have tried to solve this problem by modifying the coordination sphere via replacement of one N-donor ligand with a dimethyl sulfoxide molecule.

Among the mixed platinum(II) combinations of the PtO_2_NS type presented in the literature, only two cyclobutane-1,1-dicarboxylic platinum(II) complexes with an S-donor DMSO molecule and an N-donor molecule, namely, E-1-(9-anthryl)-3-(2-pyridyl)-2-propenone ligand (A9pyp) [23] and N6-2-methoxybenzylamino-2-chloro-9-isopropylpurine (mbip) [24], have been presented. The compound [Pt(CBDC)(DMSO)(mbip)] was a by-product of the synthesis of [Pt(CBDC)(mbip)_2_]; therefore, the authors focused only on the structural aspects of the reaction by-product. On the other hand, information on the therapeutic potential of [Pt(CBDC)(A9pyp)(DMSO)] is broader and concerns (i) cytotoxicity against CDDP-sensitive and cisplatin-resistant human ovarian cancer lines (A2780 and A2780R); (ii) intracellular accumulation; and (iii) the ability to interact with DNA. Cytotoxic studies against human ovarian carcinoma cell lines resistant to cisplatin (A2780R) revealed excellent results. The novel [Pt(CBDC)(A9pyp)(DMSO)] complex exhibits higher in vitro activity (3.2 µM) than that of cisplatin (20.4 µM). The authors stated that the presence of a less labile leaving group helps to overcome cisplatin resistance in A2780R cells. Following that line of research, we decided to design low-toxicity and effective platinum-based anticancer prodrugs with triazolopyrimidine. We obtained the four complexes [Pt(mal)(DMSO)(dmtp)], [Pt(CBDC)(DMSO)(dmtp)], [Pt(mal)(DMSO)(dptp)] and [Pt(CBDC)(DMSO)(dptp)], where dmtp is 5,7-dimethyl-1,2,4-triazolo[1,5-*a*]pyrimidine and dptp is 5,7-diphenyl-1,2,4- triazolo[1,5-*a*]pyrimidine. Finally, we found that the lipophilic platinum(II) complex with the bulky 5,7-diphenyl-1,2,4-triazolo[1,5-*a*]pyrimidine ligand, i.e., ([Pt(mal)(DMSO)(dptp)], displayed 10‑times lower in vitro toxicity than that of cisplatin against a normal cell line (BALB/3T3) and anticancer activity comparable to that of cisplatin, and indicated an in vitro cytotoxic activity against non-small cell lung carcinoma (A549) similar to that of cisplatin [25].

To continue our investigation of dicarboxylato platinum(II) compounds as potential anticancer agents, especially novel mixed platinum(II) complexes containing aromatic N-donor molecules and S-donor ligands as carrier groups (vide supra), we applied 7-isobutyl-5-methyl-1,2,4-triazolo[1,5-*a*]pyrimidine (ibmtp) and 5,7-di*tert*butyl-1,2,4-triazolo- [1,5-*a*]pyrimidine (dbtp) to prepare two new malonato platinum(II) complexes with one S-donor DMSO ligand as a non-leaving group, namely, [Pt(mal)(dbtp)(DMSO)] and [Pt(mal)(DMSO)(ibmtp)]. Additionally, we synthesized analogues to the above-mentioned complexes with cyclobutane-1,1-dicarboxylato as the leaving ligand, namely, [Pt(CBDC)(dbtp)(DMSO)] and [Pt(CBDC)(DMSO)(ibmtp)]. In the next step, novel platinum(II) complexes were comprehensively characterized by different spectroscopic methods (IR; ^1^H, ^13^C, ^15^N, ^195^Pt NMR), and the partition coefficients by the well-known shake-flask method were investigated. Moreover, we determined the in vitro antiproliferative activity and estimated the structure–lipophilicity relationship.

## 2. Materials and Methods

### 2.1. Instrumentation and Analyses

The C, H and N contents were determined using a CHN elemental analyzer, Vario Macro Elementary GMBH (Elementar Analysensysteme GMBH, Langenselbold, Germany). The IR spectra were measured on a PerkinElmer Spectrum 2000 FTIR spectrometer (PerkinElmer, Waltham, MA, USA) using KBr (400–4000 cm^−1^). The UV–Vis measurements for the determination of lipophilicity were obtained on a UVD-2950 UV–Vis spectrophotometer (Labomed, Los Angeles, CA, USA) using 1.0 cm pathlength quartz cuvettes (1.5 mL).

The NMR spectra were recorded at RT in DMF-d_7_ solutions with a Varian INOVA 500 (Varian Inc., Palo Alto, CA, USA) spectrometer operated at 499.8, 125.7, 50.6 and 107.4 MHz for ^1^H, ^13^C, ^15^N and ^195^Pt, respectively. The reference standards were TMS for ^1^H and ^13^C, CH_3_NO_2_ for ^15^N and K_2_PtCl_6_ for ^195^Pt. Gradient-enhanced IMPACT-HMBC ^1^H-(^15^N) correlation spectra [26] were optimized for a coupling constant of 6 Hz with the following experimental conditions: an acquisition time of 0.2 s, spectral windows of 6000 (F2) and 10,000 (F1) Hz, 1K complex data points, 256 time increments, 30 ms WURST-2 mixing sequence centered within a 60 ms preparation interval (ASAP^2^) and a 150° Ernst angle as the excitation pulse [27].

### 2.2. Reactants and Methods

K_2_PtCl_4_, 3-amino-1,2,4-triazole (98%), 6-methyl-2,4-heptanedione (99%), 2,2,6,6-tetramethyl-3,5-heptanedione (98%), malonic acid and cyclobutane-1,1-dicarboxlic acid were purchased from Sigma Aldrich (Merck, St. Louis, MO, USA), whereas inorganic salts and solvents of analytical grade were purchased from Avantor Performance Materials Poland S.A (Gliwice, Poland).

5,7-Disubstituted derivatives of 1,2,4-triazolo[1,5-*a*]pyrimidines were prepared according to the Bülow and Haas method by the reaction of 3-amino-1,2,4-triazolewith 6-methyl-2,4-heptanedione for ibmtp and 2,2,6,6-tetramethyl-3,5-heptanedione for dbtp [28]. Disilver malonate (Ag_2_C_3_H_2_O_4_) and disilver cyclobutane-1,1-dicarboxylate (Ag_2_C_6_H_6_O_4_) were prepared by the reaction of AgNO_3_ with malonic or cyclobutane-1,1-dicarboxylic acid by a known method [29].

### 2.3. Preparation of Dicarboxylato Platinum(II) Complexes

**Figure 1:**
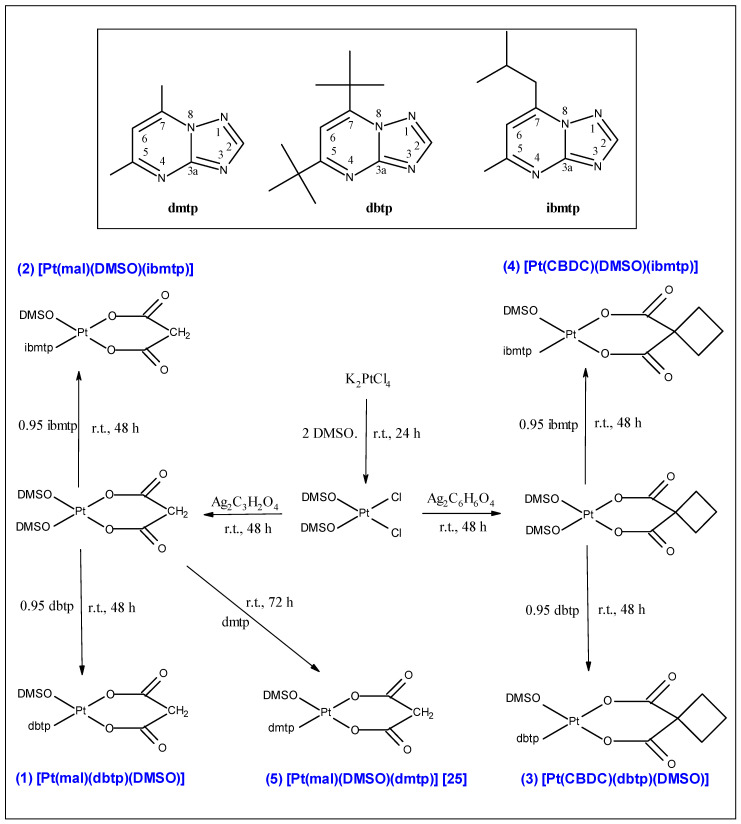
Synthesis pathways of platinum(II) complexes with 1,2,4-triazolo[1,5-*a*]pyrimidines.

The *cis*-[PtCl_2_(DMSO)_2_] used as a substrate for syntheses of complexes (**1**) [Pt(mal)(DMSO)(dbtp)], (**2**) [Pt(mal)(DMSO)(ibmtp)], (**3**) [Pt(CBDC)(DMSO)(dbtp), (**4**) [Pt(CBDC)(DMSO)(ibmtp)] and (**5**) [Pt(mal)(DMSO)(dmtp)] was prepared from K_2_PtCl_4_ by the known method [30]. The procedure of the multistep synthesis of platinum(II) complexes (**1**–**5**) is depicted in Figure 1 and described in detail as follows.

[Pt(mal)(DMSO)(dbtp)] (**1**). A solution of *cis*-[PtCl_2_(DMSO)_2_] (0.0511 g; 0.1210 mmol) in 25 mL of a 1.5:1 (*v*/*v*) mixture of ethanol and water was treated with an equivalent of silver(I) malonato (0.0365 g; 0.1147 mmol). The reaction mixture was stirred in the dark at room temperature for 48 h. Then, an equivalent of dbtp (0.0254 g; 0.1093 mmol) was added to the resulting *cis*-[Pt(mal)(DMSO)_2_] solution. The reaction mixture was further stirred under the same conditions for the next 48 h. The AgCl precipitate was removed by centrifugation and filtration. Slow evaporation of the solvent gave a pale-yellow precipitate, which was washed with acetone and diethyl ether and dried under vacuum. The (**2**–**4**) platinum(II) complexes were prepared exactly in the same way.

(**1**) (Dimethylsulfoxide-κS)(5,7-di*tert*butyl-1,2,4-triazolo[1,5-*a*]pyrimidine-κN(3))(malonato-κ^2^O,O′) platinum(II)

Yield: 0.0527 g (76.02%). ^1^H NMR (499.8 MHz, dmf-d_7_): δ = 3.51 (s, 6H, 2 × CH_3_(dmso)), 3.56 (s, 2H, CH_2_(mal)), 7.55 (s, H, H6), 9.19 ppm (s, H, H2); ^13^C NMR (125.7 MHz, dmf-d_7_): δ = 50.4 (CH_2_(mal)), 107.9 (C6), 152.6 (C3a), 152.8 (C2), 159.3 (C7), 179.8 (C5), 172.5 ppm (COO(mal)); ^15^N NMR (50.6 MHz, dmf-d_7_): δ = −105.8 (N1), −121.6 (N4), −160.2 (N8), −252.5 ppm (N3); ^195^Pt NMR (107.4 MHz, dmf-d_7_): −2609 ppm (s, PtO_2_NS). IR (KBr): 1344 (**ν_sym(OCO_—_)_**), 1541 (**ν_triazole ring_**), 1617 (**ν_pyrim ring_**), 1662 cm^−1^ (**ν_as(OCO_—_)_**).

Analysis found: C, 35.99; H, 4.79; N, 9.45%. Calculation for C_18_H_28_N_4_O_5_PtS: C, 35.59; H, 4.65; N, 9.22%.

(**2**) (Dimethylsulfoxide-*κS*)(7-isobutyl-5-methyl-1,2,4-triazolo[1,5-*a*]pyrimidine-*κN*(3))(malonato- *κ^2^O,O′*)platinum(II) monoaqua hydrate

Yield: 0.0438 g (65.46%). ^1^H NMR (499.8 MHz, dmf-d_7_): δ = 3.52 (s, 6H, 2 × CH_3_(dmso)), 3.52 (s, 2H, CH_2_(mal)), 7.59 (s, H, H6), 9.15 ppm (s, H, H2); ^13^C NMR (125.7 MHz, dmf-d_7_): δ = 42.4 (CH_2_(mal)), 114.3 (C6), 151.9 (C7), 152.3 (C3a), 153.2 (C2), 169.5 (C5), 172.9 ppm (COO(mal)); ^15^N NMR (50.6 MHz, dmf-d_7_): δ = −110.3 (N1), −120.1 (N4), −158.3 (N8), −252.1 ppm (N3); ^195^Pt NMR (107.4 MHz, dmf-d_7_): −2598 ppm (s, PtO_2_NS). IR (KBr): 1351 (**ν_sym(OCO_—_)_**), 1555 (**ν_triazole ring_**), 1636 (**ν_pyrim ring_**), 1662 cm^−1^ (**ν_as(OCO_—_)_**). 

Analysis found: C, 30.24; H, 4.52; N, 9.08%. Calculation for C_15_H_24_N_4_O_6_PtS (monohydrate): C, 30.87; H, 4.15; N, 9.60%.

(**3**) (Dimethylsulfoxide-*κS*)(5,7-di*tert*butyl-1,2,4-triazolo[1,5-*a*]pyrimidine-*κN*(3))(cyclobutane-1,1- dicarboxylato-*κ^2^O,O′*))platinum(II) monoaqua hydrate

Yield, 0.0586 g (76.52%). ^1^H NMR (499.8 MHz, dmf-d_7_): δ = 1.79, 2.83, 3.03 (6H, CH_2_(CBDC)), 3.51 (s, 6H, 2 × CH_3_(dmso)), 7.55 (s, H, H6), 9.21 ppm (s, H, H2); ^13^C NMR (125.7 MHz, dmf-d_7_): δ = 107.0 (C6), 152.7 (C3a), 153.0 (C2), 159.0 (C7), 179.7 (C5) ppm. ^15^N NMR (50.6 MHz, dmf-d_7_): δ = −106.0 (N1), −122.0 (N4), −161.0 (N8), −253.0 ppm (N3); ^195^Pt NMR (107.4 MHz, dmf-d_7_): −2607 ppm (s, PtO_2_NS). IR (KBr): 1339 (**ν_sym(OCO_—_)_**), 1542 (**ν_triazole ring_**), 1618 (**ν_pyrim ring_**), 1656 cm^−1^ (**ν_as(OCO_—_)_**).

Analysis found: C, 38.30; H, 6.16; N, 8.00%. Calculation for C_21_H_26_N_4_O_6_PtS (monohydrate): C, 37.89; H, 5.15; N, 8.42%.

(**4**) (Dimethylsulfoxide-*κS*)(7-isobutyl-5-methyl-1,2,4-triazolo[1,5-*a*]pyrimidine-*κN*(3))(cyclo- butane-1,1-dicarboxylato-*κ^2^O,O′*))platinum(II) monoaqua hydrate

Yield: 0.0498 g (69.54%). ^1^H NMR (499.8 MHz, dmf-d_7_): δ = 1.78, 2.80, 2.97 ( 6H, CH_2_(CBDC)), 3.51 (s, 6H, 2 × CH_3_(dmso)), 7.58 (s, H, H6), 9.16 ppm (s, H, H2); ^13^C NMR (125.7 MHz, dmf-d_7_): δ = 114.2 (C6), 151.9 (C7), 152.3 (C3a), 153.4 (C2), 169.4 (C5), 176.1, 176.5 ppm (COO(CBDC)). ^15^N NMR (50.6 MHz, dmf-d_7_): δ = −110.3 (N1), −119.8 (N4), −158.3 (N8), −251.7 ppm (N3); ^195^Pt NMR (107.4 MHz, dmf-d_7_): −2605 ppm (s, PtO_2_NS). IR (KBr): 1341 (**ν_sym(OCO_—_)_**), 1555 (**ν_triazole ring_**), 1636 cm^−1^ (**ν_pyrim ring_**), 1669 (**ν_as(OCO_—_)_**). 

Analysis found: C, 34.90; H, 5.10; N, 8.68%. Calculation for C_18_H_28_N_4_O_6_PtS (monohydrate): C, 34.67; H, 4.53; N, 8.98%.

(**5**) (Dimethylsulfoxide-*κS*)(5,7-dimethyl-1,2,4-triazolo[1,5-*a*]pyrimidine-*κN*(3))(malonato- *κ^2^O,O′*)platinum(II)

Details of the synthesis and spectroscopic characterization of the complex (**5**) were described previously [25].

### 2.4. Crystal Structure

Crystals of [Pt(mal)(DMSO)(dmtp)] (**5**) suitable for the diffraction experiments were obtained from the ethanol-water solution. The diffraction data were collected with the Sapphire CCD diffractometer (Oxford Diffraction, Abingdon, UK) using MoKα radiation λ = 0.71073 Å at 293(2) K by the ω-2θ method. The structure was solved by the Patterson method and refined with the full-matrix least-squares method on F^2^ with the use of the SHELX2014 [31] program package (SHECLX2014/7, G. M. Sheldrick, Georg-August-Universität Göttingen, Göttingen, Germany). Analytical absorption correction was applied (Table 1) [32]. Positions of hydrogen atoms were found from the electron density maps, and hydrogen atoms were constrained in the refinement with the appropriate riding model as implemented in SHELX. The X-ray experimental data and structure refinement for the reported structure are summarized in Table 1. The structural data have been deposited with the Cambridge Crystallographic Data Centre, deposition number CCDC 2036218. The data can be obtained free of charge from The Cambridge Crystallographic Data Centre [33].

### 2.5. Partition Coefficient

The lipophilicity (log*P*) of the platinum(II) compounds was determined using the shake–flask method [34]. At first, aqueous (0.9% *w*/*v*) sodium chloride and organic (*n*–octanol) phases were saturated for 1 week. Complexes (**2**) and (**3**) were dissolved at a concentration of 0.30 mg mL^−1^ in 2.5 mL of saturated *n*-octanol, whereas cisplatin, carboplatin and complexes (**1**) and (**4**) were dissolved in the saturated aqueous phase. An equal volume of immiscible solvent was added, and the solutions were mixed mechanically in plastic tubes for 30 min. Samples were centrifuged (6000 rpm, 15 min). After separation, the phases were analyzed by UV–Vis spectroscopy to determine the amount of the compound in each phase. The absorption values before and after shaking were compared, and based on the Lambert–Beer law, the partition coefficient in both phases for each compound was calculated to determine the log*P* values. The procedures were repeated threefold.

### 2.6. In Vitro Cytotoxicity

The in vitro antiproliferative activity against three human tumor cell lines, namely, non-small cell lung carcinoma (A549) (ATCC, Rockville, MD, USA), cisplatin-resistant human ductal breast epithelial tumor cells (T47D) (ATCC, Rockville, MD, USA) and colon cancer cells (LOVO) (ATCC, Rockville, MD, USA), and normal murine embryonic fibroblast cells (BALB/3T3) (ATCC, Rockville, MD, USA), was assessed.

Cells were plated in 96-well sterile plates (Sarstedt, Costar) at a density of 10^4^ cells per well (in 100 μL of culture medium) and incubated for 24 h. The tested compound was added in final concentrations ranging from 0.1 to 100 μg mL^−1^, and the incubation was continued for an additional 72 h. The results of in vitro cytotoxic activity are expressed as an IC_50_ (the dose of compound (in μM) that inhibits proliferation rate of tumor cells by 50%) value compared to the untreated control cells. IC_50_ values are expressed as the averages of three independently reproduced experiments. The details of this technique were described by Skehan [35]. The tests of the antiproliferative activity in vitro against both cell lines were performed with the SRB (sulforhodamine B) test using an automated microplate reader (Synergy H4 photometer, BioTek Instruments Inc., Winooski, VT, USA). Moreover, in vitro toxicity against normal murine fibroblast Balb3T3 cells was assessed. Finally, the in vitro toxicity results were compared to cisplatin, which was used as a reference at concentrations ranging from 0.01 to 10 µg/mL.

## 3. Results and Discussion

### 3.1. Multinuclear Magnetic Resonance Spectroscopy

From the application point of view, it is important to determine the environment of the central atom and the mode of the ligand binding method in platinum(II) complexes in solution. Therefore, the new dicarboxylato platinum(II) complexes were characterized in depth using multinuclear (^1^H, ^13^C, ^15^N, ^195^Pt) NMR spectroscopy, and all coordination shifts were computed in comparison to the spectra of the corresponding free ligands (Δ_coord_ = δ_complex_ − δ_ligand_). The ^1^H NMR spectra of (**1**–**4**) exhibit three of the most distinctive signals for CH_2_(mal) or CH_2_(CBDC), and H(2) and H(6) from the heterocyclic ligands. In addition, one signal corresponding to the methyl groups of the DMSO ligand was found in the range from 3.51 to 3.52 ppm (**1**–**4**). One signal at 3.56 ppm (**1**) and one at 3.52 ppm (**2**) from the mal group were observed, and signals at 1.79, 2.83 and 3.03 ppm (**3**) and 1.78, 2.80 and 2.97 ppm (**4**), all having an intensity of two, were assigned to the CBDC ring. These data were in agreement with the fact that the mal and CBDC groups act as bidentate chelating ligands through the two oxygen atoms of the carboxylato groups [36,37]. Furthermore, significant changes in the chemical shifts were observed for these hydrogen atoms from the heterocycle ligand in the aromatic region. The ^1^H NMR spectra exhibit singlets of H(6) in the range of 7.55 to 7.59 ppm (Δ_coord_ = 0.21–0.24 ppm). Notably, the largest coordination shifts were computed for H(2) from the triazole ring (maximum 0.74 ppm), which suggests the involvement of the N(1) or N(3) nitrogen atoms in the formation of Pt-N bonds. However, the ^1^H NMR data were insufficient to determine which nitrogen atoms from the 5-membered ring participate in the complexation of triazolopyrimidines to platinum(II) ions. Therefore, ^15^N–^1^H NMR spectral analysis was necessary. The signals for all nitrogen atoms were detected in the range of −105.8 to −253.0 ppm (Table 2). Based on chemical shifts, it turned out that the coordination of the trizolopyrimidine to the Pt(II) caused shielding of all nitrogen atoms. However, the highest coordination shift (Δδ^15^N_coord_ = δ^15^N_complex_ − δ^15^N_ligand_) was revealed for resonance lines of N(3) signals. The calculated coordination shifts (Δδ^15^N_coord_) ranged from −62.7 to −87.0 ppm (Table 2). These values were significantly different in comparison to those of the remaining nitrogen atoms. The strong shielding of the N(3) resonance signal clearly indicated the monodentate coordination of triazolopyrimidines to platinum(II) in solution, which was in good agreement with previous studies published for platinum(II) complexes with triazolopyrimidine derivatives [25,38,39]. According to the literature data, the absolute values of the (Δδ^15^N_coord_) for (**1**) and (**2**) complexes were lower than those found for symmetrical malonato platinum(II) complexes with two triazolopyrimidines: [Pt(mal)(dbtp)_2_] (Δδ^15^N_coord_ = −99.6 ppm), [Pt(mal)(ibmtp)_2_] (Δδ^15^N_coord_ = −108.5 ppm) [22], or triazolopyrimidine and dimethyl sulfoxide [Pt(mal)(DMSO)(dmtp)] (Δδ^15^N_coord_ = −98.3 ppm) (Table 2), [Pt(mal)(DMSO)(dptp)] (Δδ^15^N_coord_ = −100.9 ppm) [25].

To ultimately confirm the coordination mode of the triazolopyrimidine ligands to the central ion, ^13^C NMR studies were investigated. A clear pattern for the coordination shifts for complexes (**1**–**4**) was observed in the ^13^C NMR spectra (Table 3). The signals from the C(3a) atom, which was adjacent directly to the N(3) coordination site, were deshielded by approximately −4.1 to −4.6 ppm compared with those of the free ligand, whereas the C(5), C(6) and C(7) resonances shifted to the opposite side of the spectrum (0.2–3.3 ppm). This observation can be explained by the deshielding of the carbon atoms adjacent to the N(3) coordination site [40]. Furthermore, three additional signals from the malonato ligand were observed. One signal belonging to the carbon atom from CH_2_ was found at 50.4 ppm (**1**) and 42.4 ppm (**2**), and two remaining signals from the two COO groups were found for both complexes (**1)** and (**2)** at 172.5 ppm and 172.9 ppm, respectively. Likewise, the ^13^C NMR spectrum of complex (**4**) showed two signals at 176.1 and 176.5 ppm for the carbonyl carbon of coordinated carboxylate ligands. This suggests that the two carboxylato carbons are not magnetically equivalent in these complexes because one of carboxylato carbons is hindered by the proximity of the 1,2,4-triazolo[1,5-*a*]pyrimidine ring. Different environments contribute to different chemical shifts. These values were also close to the values for carboxylato carbons reported for other symmetrical [41,42] and asymmetrical platinum(II) dicarboxylato complexes [43].

**Figure 2:**
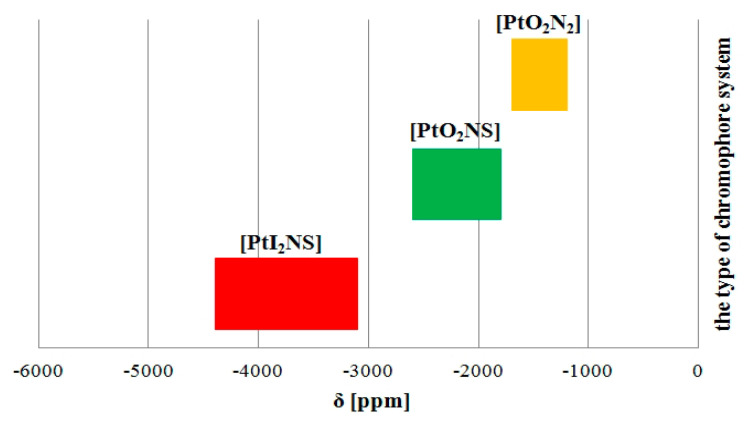
^195^Pt NMR data for different types of chromophore systems.

Nevertheless, the type of chromophore system present in the coordination sphere of (**1**–**4**) can unequivocally be determined based on their ^195^Pt resonances. ^195^Pt NMR spectra of the compounds exhibited one signal between −2598 and −2609 ppm (Table 3). There were shifts in this signal towards higher fields in comparison with that of K_2_PtCl_4_, and that could be assigned to [PtNO_2_S] coordination for platinum(II) (Table 3). The observed presence of single resonance signals indicated monodentate structures of the novel coordination complexes. These results were in good agreement with those of previous studies [23,25]. Similar results of ^195^Pt NMR data were obtained by Reedijk for chloride and cyclobutane-1,1-dicarboxylato platinum(II) complexes containing DMSO as a non-leaving group. ^195^Pt singlets for *cis*-[PtCl_2_(A9pyp)(DMSO)] and [Pt(CBDC)(A9pyp)(DMSO)] were observed at −3010 ppm [PtCl_2_NS] and −2498 ppm [PtO_2_NS] [23]. Moreover, similar values of the ^195^Pt chemical shift for complexes with two types of dicarboxylato ligands clearly confirmed that both the mal and CBDC leaving groups formed six-membered chelate rings upon complexation. The signals for previously synthesized [Pt(mal)(DMSO)(dmtp)], [Pt(CBDC)(DMSO)(dmtp)], [Pt(mal)(DMSO)(dptp)] and [Pt(CBDC)(DMSO)(dptp)] were observed in the same range of chemical shifts. Furthermore, previous studies from the Łakomska research group provide information that ^195^Pt NMR resonances of the diiodido platinum(II) complex *cis*-[PtI_2_(dbtp)_2_] were observed at much higher fields, i.e., −3261 ppm [PtI_2_N_2_], than were observed for its analogue [Pt(C_4_H_4_O_5_)(dbtp)_2_] containing dicarboxylato in the coordination sphere, −1740 ppm [PtO_2_N_2_], and for its chloride analogue *cis*-[PtCl_2_(dbtp)_2_], −2096 ppm [PtCl_2_N_2_] [39,44]. Additionally, signals from the *trans*-[PtI_2_(dbtp)(DMSO)] complex were observed at higher fields: −4389 ppm [PtI_2_NS] [40]. Moreover, the chloride analogue *trans*-[PtCl_2_(dbtp)(DMSO)] exhibited a single peak at −2955 ppm [PtCl_2_NS] [45]. Exactly the same trends were observed for the malonato platinum(II) complex [Pt(mal)(ibmtp)_2_]. ^195^Pt singlet signals were found at −1681 ppm [PtO_2_N_2_], while resonance signals of parent diiodido *cis*-[PtI_2_(ibmtp)_2_] were recorded at higher fields: −3243 ppm [PtI_2_N_2_] [22,38]. The above-mentioned data obtained by the Łakomska research group could be used to explain general trends in shielding during ^195^Pt NMR (Figure 2).

### 3.2. Infrared Studies

The IR spectra of (**1**–**4**) showed characteristic vibration bands: ν_as_(OCO—) 1662 cm^−1^ (**1**), 1657 cm^−1^ (**2**), 1656 cm^−1^ (**3**) and 1669 cm^−1^ (**4**); and the νs(OCO—) 1344 cm^−1^ (**1**), 1351 cm^−1^ (**2**), 1339 cm^−1^ (**3**) and 1341 cm^−1^ (**4**) (Table 4). The mode of carboxylato coordination to a metal ion can be determined based on the Δ values (Δ=ν_as_(OCO—)-ν_s_(OCO—)). The calculated Δ values of 318 cm^−1^ (**1**), 311 cm^−1^ (**2**), 317 cm^−1^ (**3**) and 328 cm^−1^ (**4**) compared to the Δ’ value of disodium malonato (197 cm^−1^) and of disodium cyclobutane-1,1-dicarboxylato (268 cm^−1^) suggested a monodentate coordination mode for each carboxylic acid group. Furthermore, characteristic bands of the whole pyrimidine ring skeletal vibrations from novel dicarboxylato platinum(II) complexes appeared in the range of 1541–1555 cm^−1^, whereas those of the triazolopyrimidine ring appeared in the following range: 1617–1636 cm^−1^ (Table 4). In the spectra of free ligands, the former band was found at 1530 cm^−1^ (dbtp) and 1544 cm^−1^ (ibmtp), and the latter band was found at 1615 cm^−1^ (dbtp) and 1621 cm^−1^ (ibmtp). Frequency values of absorption bands, which were assigned to relatively high frequencies in dicarboxylato complexes, clearly confirmed the coordination of dbtp and ibmtp to platinum(II) ions. Moreover, the two most characteristic bands, assigned as triazolopyrimidine ring skeletal vibrations and pyrimidine ring skeletal vibrations, were shifted by no more than 15 cm^−1^. A similar effect was already observed for different complexes with 1,2,4-triazolo[1,5-*a*]pyrimidine ligands, where N(3) coordination occurred [44,45,46]. Moreover, the strong absorption bands in the ranges of 1027–1159 cm^−1^ **(1)**, 1031–1150 cm^−1^ **(2)**, 1028–1156 cm^−1^ **(3)** and 1028–1157 cm^−1^ **(4)** indicated S-bonded DMSO, as expected for this typical soft coordination center [47,48,49,50].

Similar spectroscopic parameters were collected for PtO_2_NS complexes with 5,7-dimethyl-1,2,4-triazolo[1,5-*a*]pyrimidine (dmtp) and 5,7-diphenyl-1,2,4-triazolo[1,5-*a*]pyrimidine (dptp) [25]. The ligand coordination mode suggested in the previous work and the geometry of the coordination sphere were correct, and we could confirm this by X-ray analysis of (**5**) [Pt(mal)(DMSO)(dmtp)]. This is the first example of mixed malonato platinum(II) complex with dimethyl sulfoxide and triazolopyrimidine ligands.

### 3.3. X-ray Studies

**Figure 3:**
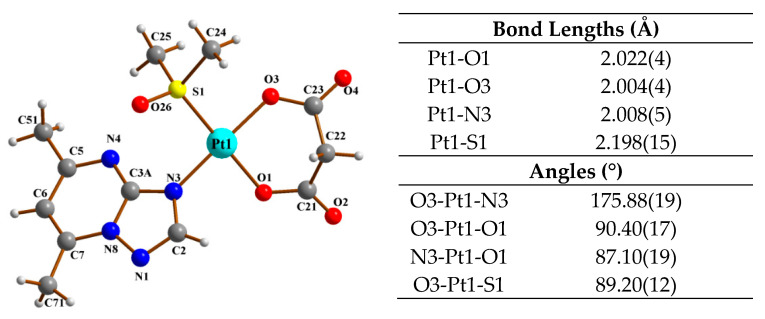
X-ray crystal structure of (**5**) [Pt(mal)(DMSO)(dmtp)] with selected bond lengths (Å) and angles (°).

Despite much effort, we were unable to obtain a single crystal of (**1**–**4**) suitable for the diffraction experiments. However, we solved the crystal structure of an analogous platinum(II) complex with another 5,7-disubstituted-1,2,4-triazolo[1,5-*a*]pyrimidine, 5,7-dimethyl-1,2,4-triazolo-[1,5-*a*]- pyrimidine (dmtp) [25]. The asymmetric region of (**5**) [Pt(mal)(DMSO)(dmtp)] consists of a single [Pt(mal)(DMSO)(dmtp)] molecule (Figure 3).

The coordination sphere PtNO_2_S had square-planar geometry. Despite the charged carboxylic groups bound to Pt, the Pt–N and Pt–O bond lengths were similar (Figure 3) and significantly shorter than the Pt1–S1 bond of 2.1979(15) Å. Such geometry was found in similar complexes reported previously [22,51]. The angular deformations within the coordination sphere were small, with cis angles varying from 87.10(19) to 93.37(15)°, while those between bonds to the ligands positioned trans were O3–Pt1–N3 175.88(19)° and O1–Pt1–S1 178.81(13)°. The O3–Pt1–O1 angle between the coordination bonds formed by malonate was 90.40(17)°. For the NO_2_S coordination sphere, the rms deviation of atoms from the best plane was 0.039 Å, and the Pt1 deviation from that plane was −0.018 Å.

The valence geometry of dmtp was typical for such systems. The tilt of the dmtp ring system relative to the coordination plane might be described with a C3A-N3-Pt1-O1 torsion angle of −127.0(5)°. The dihedral angle between the best plane of the dmtp ring system and the NO_2_S coordination plane was 53.5(1)° with rotation around the Pt1–N3 bond in a direction minimizing the dmtp interactions with O26 of DMSO. In this orientation, interactions were found between the C25 methyl group of DMSO and the C51 methyl group of dmtp.

In the reported complex, the malonate ion was a bidentate ligand. The presence of three single bonds allowed for the relaxation of possible strain by rotation of both carboxylic groups relative to the C21-C22-C23 fragment, with the dihedral angles between this fragment and O1-C21-O2-C22 and O3-C23-O4-C22 and the best planes being 33.6(4)° and 50.3(7)°, while that between the two carboxylic groups was 83.9(4)°. The six-membered chelate ring formed by malonate (Figure 3) had a boat conformation.

The valence geometry of DMSO was typical. The orientation of this ligand relative to the coordination plane was reflected in the N3-Pt1-S1-O26 and N3-Pt1-S1-C25 torsion angles of 24.6(3)° and −102.1(3)°, respectively.

### 3.4. Lipophilicity

**Figure 4:**
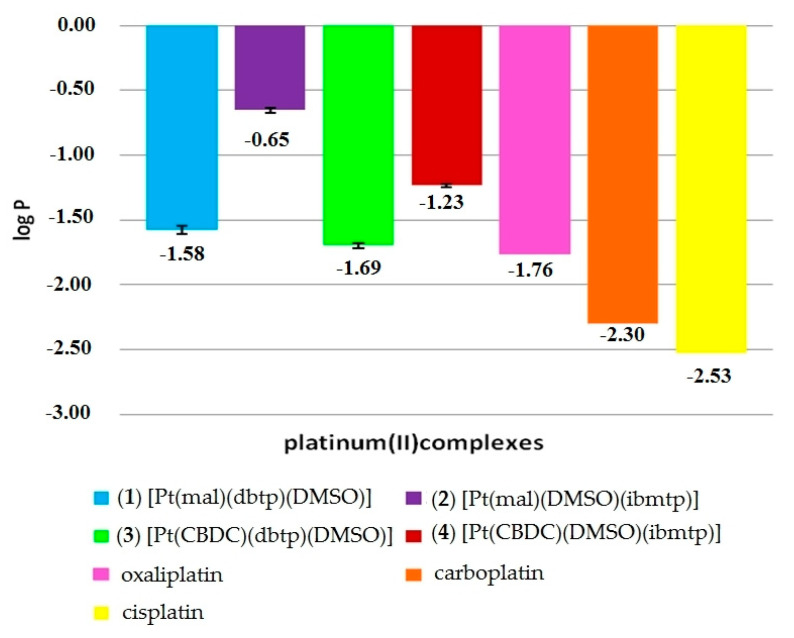
Lipophilicity of platinum(II) complexes.

The lipophilicity of the investigated compounds increased in the order of (**1**) < (**3**) < (**4**) < (**2**) (Figure 4). These values can be compared with data available in the literature for currently used inorganic platinum(II) drugs, namely, cisplatin (−2.53), carboplatin (−2.30) and oxaliplatin (−1.76) [52]. It is important to note that that all studied platinum(II) complexes exhibited increased lipophilicity in comparison to those of all three international anticancer drugs.

Additionally, the study indicated that the log*P* for complexes (**1**–**4**) correlated with the type of dicarboxylate ligand (leaving ligands). The obtained results confirmed that the malonato platinum(II) complexes [Pt(mal)(dbtp)(DMSO)] (−1.58) and [Pt(mal)(DMSO)(ibmtp)] (−0.65) were more lipophilic than their cyclobutane-1,1-dicarboxylate analogues [Pt(CBDC)(dbtp)(DMSO)] (−1.69) and [Pt(CBDC)(DMSO)(ibmtp)] (−1.23).

According to the literature, cellular uptake is a commonly known factor influencing drug efficacy. Additionally, cellular uptake of platinum(II) complexes by passive diffusion correlates with their lipophilicities [53]. Lipophilicity has been widely investigated in quantitative structure–activity relationships (QSARs) for bioavailability, transmembrane transport or cellular drug accumulation [54]. Currently, the lipophilicity of drugs is related to their ability to cross cell membranes by passive diffusion. Moreover, this parameter can be correlated with the relative solubility of the molecule in lipid bilayers and the aqueous environment in cells. Increasing the lipophilic character of platinum(II) complexes facilitates drug accumulation via the lipid bilayer of the cell membrane. However, such high lipophilicity simultaneously decreases aqueous solubility, which finally impedes drug application [55].

Generally, platinum(II) complexes are hydrophilic, with negative log*P*o/w values [56]. However, the addition of large nonpolar ligands such as 1,2,4-triazolo[1,5-*a*]pyrimidine derivatives can increase lipophilicity. Following the previous work of the Łakomska research group, the [Pt(mal)(ibmtp)_2_] (0.80) and [Pt(mal)(dbtp)_2_] (2.07) complexes possessed positive log*P* values [22], which indicated their relatively high lipophilicity. The neutral ligands imparted lipophilic character to the dicarboxylato platinum(II) complexes; however, these ligands also reduced their water solubility. On the basis of these findings, we decided to modify the structure of dicarboxylato platinum(II) complexes by introducing the DMSO molecule as a S-donor non-leaving group.

### 3.5. In Vitro Antiproliferative Activity

The in vitro cytotoxicity of the studied platinum(II) complexes (**1**–**4**) against A549, LoVo and T47D cells is summarized in Table 5. The obtained results (Table 5) clearly indicated that the platinum(II) complexes with the dbtp molecule as the non-leaving group possessed higher in vitro cytotoxicity than that of platinum(II) analogues with the ibmtp molecule. Additionally, it could be detected that both dicarboxylato leaving groups possessed comparable influences on the cytotoxicity of the platinum(II) complexes. This can be caused by the fact that both mal and CBDC ligand chelation produced six-membered rings with platinum(II) ions, which provide the same stability and activity. Among all dicarboxylato platinum(II) complexes, (**3**) [Pt(CBDC)(dbtp)(DMSO)] exhibited significant in vitro cytotoxic activity against A549 and T47D cancer cells. These results confirmed that complexes with bulky triazolopyrimidine ligands exhibit relatively high activity against cancer cell lines, which was in good agreement with previous studies [22,25]. Additionally, based on these results, there was a clear correlation between lipophilicity and in vitro activity. The most lipophilic complexes, namely, (**1**) [Pt(mal)(dbtp)(DMSO)] and (**3**) [Pt(CBDC)(dbtp)(DMSO)], showed better antiproliferative activity than that of the more hydrophilic complexes. Due to the highly toxic activity of platinum compounds against normal cells, there was a requirement to investigate their antiproliferative activity against normal cells. Thus, the in vitro toxicity towards normal mouse embryonic fibroblast cells was estimated. The (**3**) [Pt(CBDC)(dbtp)(DMSO)] complex revealed lower toxicity to BALB/3T3 cells than cisplatin.

**Figure 5:**
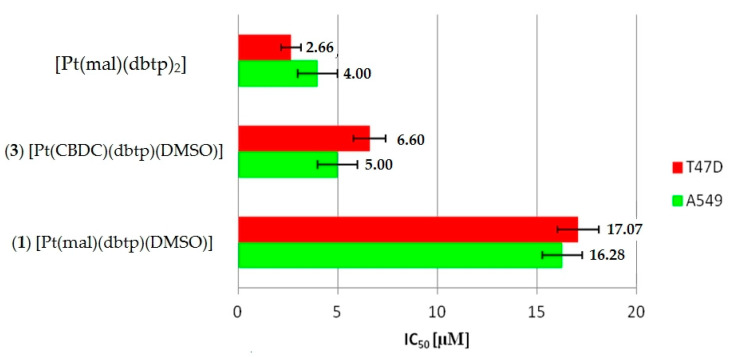
Influences of carrier ligand and leaving group on in vitro cytotoxicity.

Our previous [22] and current in vitro results agree with two main mechanistic assumptions concerning the design and synthesis of novel platinum(II) drugs. First, replacement of the leaving group influenced the activity by pharmacological properties. Second, adding an appropriate carrier group to a transition metal can grant more favorable DNA binding properties [57]. Such a tendency can be observed among the activities of our dicarboxylato platinum(II) complexes (Figure 5). On the one hand, substitution of the mal ligand by the CBDC group increased the activity. On the other hand, substitution of one of the 5,7-di*tert*butyl-1,2,4-triazolo[1,5-*a*]pyrimidine carrier ligands by the DMSO molecule slightly decreased the in vitro cytotoxicity. This can be explained by the relatively low capacity of interaction between bases in DNA and DMSO. Bulky ligands such as 5,7-di*tert*butyl-1,2,4-triazolo[1,5-*a*]pyrimidine possess a higher capacity to increase the intercalation with DNA than the smaller DMSO molecules. Such a tendency is revealed in results against non-small cell lung carcinoma (A549) and breast cancer (T-47D) cells.

The results once again emphasize [22,25] the significant influences of type of triazolopyrimidine ligand (especially the types of substituent at positions 5 and 7) and the composition of the coordination sphere on the formation of cytotoxic platinum(II) complexes. These things are very useful in the design of new antitumor drugs, but in the future it will be necessary to enrich understanding with advanced research aimed at understanding their mechanisms of action.

Additionally, we analyzed the in vitro antiproliferative activity of the most promising compound (**3**) [Pt(CBDC)(dbtp)(DMSO)], cisplatin and our previously described platinum(II) complex with another 5,7-disubstituted-1,2,4-triazolo[1,5-*a*]pyrimidine (dptp), [Pt(mal)(DMSO)(dptp)], where dptp is 5,7-diphenyl-1,2,4-triazolo[1,5-*a*]pyrimidine [25]. Within this group of compounds, [Pt(mal)(DMSO)(dptp)] exhibited the most selective cytotoxicity in vitro (IC_50_ = 10.1 ± 10.7 µM). This complex showed similar anticancer activity to that of cisplatin (IC_50_ = 9.00 ± 1.1 µM) and (**3**) [Pt(CBDC)(dbtp)(DMSO)] (IC_50_ = 5.00 ± 0.9 µM) towards the A549 cell line. Moreover, the most significant advantage of this complex was that it exhibited low toxicity (IC_50_ = 37.1 ± 7.0 µM) towards the BALB/3T3 normal cell line in comparison with that of cisplatin (IC_50_ = 3.20 ± 1.5 µM) and complex (**3**) (IC_50_ = 10.19 ± 13.2 µM). For that reason, we can suggest that the use of 5,7-diphenyl-1,2,4-triazolo[1,5-*a*]pyrimidine as an N-donor ligand as an appropriate way to design selective anticancer platinum(II) compounds.

## 4. Conclusions

Motivated by the success of cisplatin, carboplatin and oxaliplatin in cancer treatments, we made efforts to investigate new dicarboxylato platinum(II) complexes. Here, we report a series of novel, mixed dicarboxylato platinum(II) complexes, namely, the two mononuclear malonato platinum(II) complexes - [Pt(mal)(DMSO)(dbtp)] (**1**) and [Pt(mal)(DMSO)(ibmtp)] (**2**); and the two mononuclear cyclobutane-1,1-dicarboxylato complexes - [Pt(CBDC)(DMSO)(dbtp)] (**3**) and [Pt(CBDC)(DMSO)(ibmtp)] (**4**), which were successfully synthesized and fully characterized using ^1^H, ^13^C, ^15^N and ^195^Pt NMR and IR spectroscopy. Considering the IR spectra of the complexes along with the results obtained from experimental and computational studies, it was concluded that each carboxylic acid group exhibited a monodentate coordination mode. Significant ^15^N NMR shielding (62–87 ppm) was observed for the N(3) atom. These data indicated that coordination of the investigated 1,2,4-triazolo[1,5-*a*]pyrimidines to platinum(II) ions occurs via N(3). Additionally, the results obtained from NMR studies confirmed the existence of all novel complexes in solution. This evidence is highly essential with respect to the biological applications of these platinum(II) complexes (**1**–**4**), who were more lipophilic than the most common anticancer drugs, such as cisplatin and the dicarboxylato analogues carboplatin and oxaliplatin. Finally, among all dicarboxylato platinum(II) complexes, [Pt(CBDC)(dbtp)(DMSO)] (**3**) exhibited significant in vitro cytotoxic activity against A549 and T47D cancer cell lines.

In summary, the [Pt(CBDC)(DMSO)(dbtp)] (**3**) complex was shown to be a promising candidate as an anticancer drug due to the following factors:

**(i)**  Relevant cytotoxic activity against A-549 (non-small cell lung carcinoma) and T47D (breast cancer) cells;
**(ii)**  Better selectivity against A549 and T47D than cisplatin;
**(iii)**  Lower toxicity against normal BALB/3T3 cells than that of cisplatin.

## Figures and Tables

**Table 1:** Crystal data and structure refinement for (**5**) [Pt(mal)(DMSO)(dmtp)].

Parameter	Result
Empirical formula	C_12_H_16_N_4_O_5_PtS
Formula weight	523.44
Temperature; K	293(2)
Wavelength; Å	0.71073
Crystal system	Orthorhombic
Space group	Pbca
Unit cell dimensions; Å,	a = 11.7627(4)
	b = 8.8447(3)
	c = 30.8525(11)
	α
	β
	γ
Volume; Å^3^	3209.82(19)
Z	8
Density (calculated); Mg/m^3^	2.166
Absorption coefficient; mm^−1^	8.903
F(000)	2000
Crystal size; mm	0.331 × 0.217 × 0.022
Theta range for data collection	2.177 to 28.678°.
Index ranges	−10 ≤ h ≤ 15, −11 ≤ k ≤ 11, −34 ≤ l ≤ 38
Reflections collected	20604
Independent reflections	3785 [R(int) = 0.0540]
Completeness to theta	25.242°, 99.8%
Absorption correction	Analytical
Max. and min. transmission	0.8291 and 0.1618
Refinement method	Full-matrix least-squares on F^2^
Data/restraints/parameters	3785/0/208
Goodness-of-fit on F^2^	1.060
Final R indices [I > 2sigma(I)]	R1 = 0.0395, wR2 = 0.0760
R indices (all data)	R1 = 0.0601, wR2 = 0.0802
Largest diff. peak and hole; e.Å^−3^	2.06 d −3.949

**Table 2:** ^1^H and ^15^N NMR chemical shifts (δ) of platinum(II) complexes [in ppm].

Compounds	^1^H		^15^N			
	H(2)	H(6)	N(1)	N(3)	N(4)	N(8)
(**1**) [Pt(mal)(dbtp)(DMSO)]	9.19 (+0.39)	7.55 (+0.24)	−105.8 (−0.6)	−252.5 (−86.2)	−121.6 (−5.2)	−160.2 (−3.7)
(**2**) [Pt(mal)(DMSO)(ibmtp)]	9.15 (+0.73)	7.59 (+0.22)	−110.3 (−4.3)	−252.1 (−62.7)	−120.1 (−6.0)	−158.3 (−1.2)
(**3**) [Pt(CBDC)(dbtp)(DMSO)]	9.21 (+0.41)	7.55 (+0.24)	−106.0 (−0.4)	−253.0 (−85.7)	−122.0 (−4.8)	−161.0 (−2.2)
(**4**) [Pt(CBDC)(DMSO)(ibmtp)]	9.16 (+0.74)	7.58 (+0.21)	−110.3 (−4.3)	−251.7 (−87.0)	−119.8 (−6.3)	−158.3 (−1.2)
(**5**) [Pt(mal)(DMSO)(dmtp)] [25]	9.14 (+0.55)	7.58 (+0.39)	−109.7 (+2.2)	−252.2 (−98.3)	−120.8 (−5.0)	−157.4 (−2.1)

**Table 3:** ^13^C and ^195^Pt NMR chemical shifts (δ) of platinum(II) complexes [in ppm].

Compounds	C(2)	C(3a)	C(5)	C(6)	C(7)	Pt
(**1**) [Pt(mal)(dbtp)(DMSO)]	152.8 (−0.1)	152.6 (−4.1)	179.8 (+3.1)	107.9 (+3.3)	159.3 (+1.4)	−2609 (−985)
(**2**) [Pt(mal)(DMSO)(ibmtp)]	153.2 (+2.0)	152.3 (−4.6)	169.5 (+0.2)	114.3 (+0.9)	151.9 (+0.7)	−2598 (−974)
(**3**) [Pt(CBDC)(dbtp)(DMSO)]	153.0 (+0.1)	152.7 (−4.2)	179.7 (+3.2)	107.0 (+4.2)	159.0 (+1.7)	−2607 (−983)
(**4**) [Pt(CBDC)(DMSO)(ibmtp)]	153.4 (+1.8)	152.3 (−4.6)	169.4 (+0.3)	114.2 (+1.0)	151.9 (+0.7)	−2605 (−981)

**Table 4:** IR spectroscopic data for platinum(II) complexes (in cm^−1^).

Compounds	ν_as(OCO_—_)_	ν_pyrim. ring_	ν_triazole ring_	ν_sym(OCO_—_)_	Δ	Δ’
(**1**) [Pt(mal)(dbtp)(DMSO)]	1662	1617	1541	1344	318	197
(**2**) [Pt(mal)(DMSO)(ibmtp)]	1662	1636	1555	1351	311	
(**3**) [Pt(CBDC)(dbtp)(DMSO)]	1656	1618	1542	1339	317	268
(**4**) [Pt(CBDC)(DMSO)(ibmtp)]	1669	1636	1555	1341	328	

**Table 5:** In vitro antiproliferative activity of platinum(II) complexes (in µM).

Compounds	IC_50_^a^			
	A549	LoVo	T47D	BALB/3T3
(**1**) [Pt(mal)(dbtp)(DMSO)]	16.28 ± 9.9	27.92 ± 1.0	17.07 ± 10.5	30.25 ± 22.6
(**2**) [Pt(mal)(DMSO)(ibmtp)]	57.81 ± 13.5	38.35 ± 1.8	42.6 ± 19.0	45.95 ± 40.7
(**3**) [Pt(CBDC)(dbtp)(DMSO)]	5.00 ± 0.9	20.02 ± 2.8	6.60 ± 4.8	10.19 ± 6.2
(**4**) [Pt(CBDC)(DMSO)(ibmtp)]	57.29 ± 11.3	41.19 ± 1.6	21.87 ± 17.2	73.40 ± 29.7
cisplatin	9.00 ± 1.1	0.84 ± 0.3	3.11 ± 1.2	3.20 ± 1.5

^a^ The values are averages of three independent determinations.
